# Assessment of gestational diabetes mellitus knowledge, attitudes, and practices and associated factors among pregnant women at a district hospital in Coastal Kenya

**DOI:** 10.1186/s41182-024-00630-3

**Published:** 2024-10-22

**Authors:** Nanyangwe Siuluta, Miho Sato, Le Khac Linh, Violet Wanjihia, Mwatasa Salim Changoma, Nguyen Tien Huy, Satoshi Kaneko

**Affiliations:** 1 Graduate School of Tropical Medicine and Global Health (TMGH), Nagasaki University, Nagasaki, Japan; 2 College of Health Sciences, Vin University, Hanoi, Vietnam; 3 Center for Public Health Research, Kenya Medical Research Institute, Nairobi, Kenya; 4 Nagasaki University Institute of Tropical Medicine-Kenya Medical Research Institute (NUITM-KEMRI) Project, Kwale, Kenya

## Abstract

**Background:**

Gestational diabetes mellitus (GDM) is any degree of glucose intolerance first recognized during pregnancy. GDM awareness among pregnant women translates into GDM prevention and early diagnosis.

**Objective:**

To establish the underlying factors influencing GDM Knowledge Attitude and Practices (KAP) among pregnant women at Kinango District Hospital.

**Method:**

An explanatory mixed-methods design was implemented by initially assessing GDM KAP quantitatively [QUAN], followed by the qualitative [qual] exploration of contextual factors behind quantitative results. In the [QUAN] strand, 354 pregnant women were interviewed from January to February 2019. Thereafter, in the [qual] strand, key informant interviews were conducted among four pregnant women and three healthcare workers; a focus group discussion was held among nine pregnant women, from May to June 2019. STATA V15 software package was used to analyze the quantitative data. Qualitative data were analyzed manually using thematic analysis.

**Result:**

Among 354 pregnant women, 29.0% were knowledgeable, 46.98% had good attitude and 60.17% had good practice. Attending at least one antenatal clinic visit and having heard about diabetes mellitus were associated with good GDM attitude and practices. Among the knowledgeable pregnant women, one-third (33.33%) obtained GDM information from a health facility. Discussions with pregnant women and the interviews with healthcare workers highlighted that daily health talks during antenatal clinic, included GDM and diabetes mellitus information. Furthermore, attendance of at least one antenatal clinic visit was low (85.88%), among pregnant women (87.86%) who were expected to have attended at least one antenatal clinic visits.

**Conclusion:**

Despite low GDM knowledge, pregnant women had relatively good GDM attitudes and good GDM practices. Daily health talks conducted during antenatal clinic as well as indigenous knowledge among pregnant women, influenced the latter. Hence, GDM information dissemination needs to be enhanced for the improvement of GDM KAP among pregnant women for GDM prevention.

## Introduction

Gestational diabetes mellitus (GDM) is any degree of glucose intolerance of variable degree with onset or first recognition during pregnancy [1]. The global prevalence of hyperglycemia in pregnant women between 20–49 years was estimated to be 16.9%, affecting 21.3 million live births and more than 90% of cases were estimated to occur in low and middle-income countries (LMIC) [2]. Currently, diabetes mellitus (DM) affects more than 250 million people worldwide and is expected to affect over 380 million by 2025 [3]. A systematic review of 14 studies conducted in six African countries (i.e., Ethiopia, Morocco, Mozambique, Nigeria, South Africa, and Tanzania), reported a prevalence ranging from as low as 0% to as high as 13.9% [4]. Very few studies have been conducted on GDM prevalence in Kenya, a study at Kenyatta University Hospital, which is a leading national referral hospital, reported a GDM prevalence of 11.6% [5]. The World Health Organization (WHO) estimates the prevalence of DM in Kenya at 3.3% and it is predicted to rise to 4.5% in 2025 [6]. Urbanization in Africa may be a contributing factor to the evolving problem of non-communicable diseases (NCDs) [7, 8]. Hence, GDM has become a public health concern, as maternal health offers a window of opportunity to improve health and prevent the intergenerational transmission of NCDs, particularly diabetes mellitus.

The importance of GDM in impacting diabetes prevalence has been stressed [9]. Maternal health is crucial for women's well-being and future generations [10]. Efforts should focus on preventing factors influencing maternal mortality to achieve Sustainable Development Goal targets by 2030 [11–13]. While interventions usually address direct causes like hemorrhage, indirect causes like NCDs have been overlooked. Low-income countries lack resources to manage GDM-related complications, emphasizing the need to invest in GDM prevention through lifestyle changes and education [10]. The large numbers of women with hyperglycemia in LMICs are alarming as these countries are the least well-equipped to provide interventions for the management of complications related to GDM and this may further disadvantage affected women and infants [12]. In order to provide baseline information for the implementation of a GDM prevention program, a knowledge, attitude and practices (KAP) survey is deemed necessary [14]. Studies across various countries have shown inadequate knowledge regarding GDM among pregnant women but relatively higher awareness about diabetes in general [14–18]. However, high knowledge levels regarding DM have been reported too [19–21].

A study by Kiberenge et al. revealed regional differences in DM knowledge in Kenya, with the Coast Province having the lowest level of DM knowledge [7]. Regional disparities in diabetes awareness highlight challenges faced in Kenya [6]. In addition, previous studies have assessed attitudes and practices toward DM and GDM that vary among clients. Poor attitudes among clients have been recorded by studies [7, 14]. However, another study has reported good attitudes among clients too [21]. Good practice levels among clients have been reported [7, 21] as well as poor practices [14]. The factors that elevate the risk of GDM during pregnancy include obesity, lack of physical activity, being of advanced maternal age, having multiple previous pregnancies, a family history of type 2 diabetes mellitus. Additionally, a history of a large baby in a previous pregnancy, a previous occurrence of GDM, and the presence of polycystic ovarian syndrome are also identified as risk factors [22, 23]. In view of very few KAP studies regarding GDM in Kenya, the author of the study conducted at Kenyatta University Hospital recommended more studies to be carried out in Kenya with regard to knowledge, attitudes, and practices about GDM among the general population and among healthcare workers [5]. The paucity of data on GDM KAP, prompts the need for more research among the general population and healthcare workers. This study at Kinango District Hospital aimed to fill this knowledge gap, by assessing GDM KAP levels and associated risk factors among pregnant women. The findings aim to guide primary preventive measures, preventing GDM occurrence and subsequent transmission of diabetes across generations in rural Coastal Kenya.

## Materials and methods

### Study design, period, site, and population

This study was conducted at the Antenatal Clinic and Maternity Ward at Kinango District Hospital located in Kwale County, Kenya. This is a district hospital under the Kenyan Ministry of Health, located in Kwale County of Kenya, which offers both Antenatal Care (ANC) and Postnatal Care (PNC) services, and covers a population of 22,392 women in the reproductive age group. A mixed-methods study using an explanatory sequential design [24–26] was adopted. The quantitative method was implemented first from January 2019 to February 2019. During this period, we assessed the level of GDM KAP, as well as the factors associated with GDM KAP among the pregnant women seen at Kinango District Hospital. During this part of the survey, an analytical cross-sectional quantitative study was adopted. Thereafter, the qualitative methods were implemented from May 2019 to June 2019. This strand was selected in order to expand or confirm the findings of the quantitative data of the first strand by interviewing key informants including pregnant women and the healthcare workers at Kinango District Hospital.

Regarding the study population, for the quantitative component, 354 pregnant women meeting the inclusion criteria were sampled and interviewed after obtaining written informed consent. Pregnant women of reproductive age group that could give written consent (aged 18–45 years) of any gestational age were eligible for participation. The participants were recruited by convenience sampling, because they were interviewed as they reported to the facility for an antenatal clinic or maternity ward visit. However, pregnant women who were not willing to be interviewed were respected and excluded from our study. For the qualitative component, participants were selected via purposive sampling. One focus group discussion (FGD) was conducted with nine QUAN participants who agreed to take part in the FGD. Thereafter, key informant interviews (KIIs) were conducted with four pregnant women, who were selected among the FGD participants. Of the four, two pregnant women who contributed the most and two who contributed the least were selected. In addition, one midwife, one clinical officer, and one senior registered nursing officer were also interviewed individually as key informants.

### Variables

For the analytical study component involving pregnant women, the dependent variables were knowledge, attitude, and practice levels while the independent variables were the baseline characteristics of the participants, i.e., age, gestational age, parity, highest level of education, source of income, ever heard about DM, family history of DM, history of previous stillbirth, birth weight of previous child, history of previous Cesarean section (C/S) and number of ANC visits.

### Sample size determination

A total of 354 pregnant women were selected using the formula below, to calculate the adequate sample size using cross-sectional study design [27]:

$$n = Z^{2} P\left( {1 - P} \right)/d^{2} .$$

*Z* was the statistic corresponding to the level of confidence, in this case 95% confidence interval. While *P* was the level of good knowledge in those that had attained at least primary education (that was obtained from previously published studies), in this case, 14% [7]; and *d* was the precision (corresponding to effect size), in this case, 5%.

### Data collection

For the quantitative component pregnant women were asked questions using face-to-face interviewer-administered standardized questionnaires. The interviews were administered by a researcher-administered questionnaire via Open Data Kit on an Android phone. The research assistant was a native of Kenya, fluent in Swahili (the local language) and English. The questionnaires were designed by the principal investigator (PI) after reviewing various literature. For the pregnant women interview, the first part of the questionnaire covered the general information of the pregnant women. General information included participant ID, interviewer name, date of survey, hospital name, area name, participant name, and participant phone number.

This information was collected to facilitate contact for the qualitative interviews.

The second part covered the socio-demographic information including age, gestational age, parity, highest level of education, source of income, having heard about diabetes mellitus, and family history of diabetes mellitus. The third part assessed the past medical history of the clients, while the fourth part assessed the level of knowledge pregnant women had regarding GDM. Furthermore, pregnant women were asked about GDM risk factors, signs and symptoms, control and management, and complications. Respondents answered “Yes” “No” or “I don’t Know”. The fifth part assessed the attitude of the respondents towards GDM. A five-point Likert scale was used to assess attitude (i.e., 5 = strongly agree, 4 = agree, 3 = neutral, 2 = disagree, 1 = strongly disagree) towards GDM [28]. The sixth part covered the practices regarding GDM. A five-point Likert scale was also used to assess practices (i.e., 5 = very frequent, 4 = frequent, 3 = not sure, 2 = less frequent, and 1 = not at all) [28]. The seventh part assessed the experiences of pregnant women regarding GDM, most especially, the common GDM information source. The questionnaire was pretested on five pregnant women to clarify the importance of various components, assess the suitability of the content, and establish the appropriate flow of questions.

After assessing GDM KAP quantitatively, the qualitative exploration of contextual factors behind quantitative results was conducted. KIIs were conducted on four purposively selected pregnant women, three healthcare workers, and a FGD with nine pregnant women. The research assistant interviewed the pregnant women while the principal investigator (PI), a Zambian native and fluent in English, interviewed the healthcare workers. Each KII was conducted with a semi-structured interview guide. Field notes were also always taken during the interviews. KIIs were conducted iteratively on two separate visits (for over an hour each), to get an in-depth understanding of the local context; KIIs were conducted by the research assistant, who is a native speaker of Swahili, under close supervision of the PI. The research assistant also facilitated the FGD; she then made a verbatim transcription of the FGD recordings in English for the PI to understand. The PI read all the transcripts numerous times, asking for clarifications from the research assistants regularly. KIIs and a FGD with pregnant women were conducted to get an insight into the GDM experiences and challenges among pregnant women that they faced during their visit to Kinango District Hospital. For the KIIs with healthcare workers, the PI conducted the interviews in English. These KIIs were conducted with healthcare workers to get an insight into the management of pregnant women as well as an in-depth understanding of what challenges healthcare workers in the local setup face in managing patients according to the Kenyan National Guidelines for Quality Obstetric and Perinatal Care (KNGQOPC) formulated by the Kenyan Ministry of Health [29].

All questionnaires, Information sheets, and informed consent were prepared in English language and then translated to Swahili (local language in Kenya) via forward translation.

### Scoring

For the analytical component of the cross-sectional study, the mean scores of GDM KAP were calculated [28]. To calculate the mean score of knowledge from respondents, those who answered “Yes” were considered as correctly answered; “No” and “I don’t know” were considered as incorrectly answered. Those who scored the mean and above were classified as knowledgeable, while those who scored below the mean score of knowledge questions were classified as not knowledgeable [28]. Furthermore, a five-point Likert scale was used to measure the attitude and practice levels [28]. All pregnant women’s attitudinal and practice answers were computed to obtain a total score and the mean score was calculated. Pregnant women who scored the mean and above were considered to have good attitudes and good practices, respectively; while those who scored below the mean had poor attitudes and poor practices [28].

### Data management and analysis

For the quantitative component of the study, the completed questionnaires were checked for completeness and consistency by the PI and research assistant. The data were then sent to the Nagasaki University Eco-Epidemiology Laboratory server. Data were then converted from CSV format into an Excel sheet. Thereafter, data were imported into STATA V15 software package. Data were explored, cleaned, and coded accordingly. For the analytical quantitative survey involving pregnant women, univariate analysis was conducted with the use of frequency distribution curves, and histograms and summarized using means, mode, median, standard deviations, and quartiles. Then binary logistic regression analysis was conducted to see the independent effect of predictor variables on the dependent variable. Predictors that had a statistically significant association with bivariate analysis were entered into the multivariate logistic regression analysis model to find the final predictors of KAPs regarding GDM, after controlling for other independent variables. The odds ratio and 95% confidence interval were calculated; a *P*-value of less than or equal to 0.05 was considered statistically significant. Finally, results were summarized and presented in tables and graphs; thereafter, they were described in text form and interpreted accordingly.

For the qualitative component of this study, the recorded data (transcribed verbatim) in local dialect (Swahili) were translated to English via forward translation. This was followed by thematic analysis [30] using Microsoft Word. A semantic approach, focusing on the explicit meaning of the data was chosen [30]. Transcripts were reviewed independently by the PI, who followed the phases outlined by Braun and Clarke [30]. Phase 1 involved familiarization with the data, reading and re-reading of the transcripts, and note-taking of ideas. Phase 2 involved systematically generating initial codes across the entire data set. Phase 3 involved collecting the codes into potential themes. Phase 4 involved reviewing the themes and ensuring that they were relevant in relation to both the coded extracts and the entire data set. Phase 5 involved refining the themes to ensure that they provided a clear reflection of the overall story portrayed in the data set. Finally, in Phase 6, the PI transformed the analysis into an interpretive piece of writing by using vivid and compelling extract examples that related to the themes, research question, and literature [30]. As such, thematic categories derived from the qualitative data were used to give an in-depth explanation of the quantitative data to provide rich detail that facilitated an understanding of the underlying factors influencing the GDM KAP at Kinango District Hospital.

### Ethics approval and consent to participate

Ethical approval for this study was granted by the Ethics Committee of the School of Tropical Medicine and Global Health (TMGH), Nagasaki University, and from the Scientific Ethics Review Unit (KEMRI/SERU) of the Kenya Medical Research Institute (KEMRI). This study was approved under the Academic Cooperation Agreement made between KEMRI and Nagasaki University, and it was conducted by ethical regulations and laws of Kenya and the declaration of Helsinki of 1964. All participants were informed about the purpose of the study and signed informed consent before participation.

## Results

### Baseline characteristics of pregnant women attended to at Kinango District Hospital

Among pregnant women seen at Kinango District Hospital, 67% of them heard about DM. The attendance of at least one ANC visit prior to the study interview day was 85.88% (Table 1).

**Table 1:** Baseline characteristics of pregnant women (*N* = 354) at Kinango District Hospital, 2019

Maternal variable	Number	Proportion (%)
*Age (years)*		
< 24	149	(42.09)
25–34	160	(45.20)
35–44	42	(11.86)
> 44	3	(0.85)
*Gestational age*		
Less than 24 weeks	43	(12.15)
24–28 weeks	144	(40.68)
29–42 weeks	167	(47.18)
*Parity*		
Zero	69	(19.49)
One to two	144	(40.68)
Three to four	90	(25.42)
Five or more	51	(14.41)
*Highest level of education*		
Never attended school	123	(34.75)
Primary	190	(53.67)
Secondary	26	(7.34)
Post-secondary	15	(4.24)
*Source of income*		
Housework/informal employment	291	(82.20)
Business	40	(11.30)
Agriculture	10	(2.82)
Formal employment	13	(3.67)
*Ever heard about diabetes mellitus*		
Yes	238	(67.23)
No	116	(32.77)
*Family history of diabetes mellitus*		
Yes	37	(10.45)
No	317	(89.55)
*History of previous still birth*		
Yes	11	(3.11)
No	343	(96.89)
*Birth weight of previous child*		
Low birth weight (< 2.5 kg)	15	(4.24)
Normal birth weight (2.5-4 kg)	252	(71.19)
High birth weight (> / = 4 kg)	3	(0.85)
No Previous birth weight	84	(23.73)
*History of previous Cesarean section*		
Yes	15	(4.24)
No	339	(95.76)
*Number of past antenatal care visits*		
Zero	50	(14.12)
One	55	(15.54)
Two	57	(16.10)
Three	100	(28.25)
Greater than or equal to 4	92	(25.99)

### Knowledge

#### Knowledge level of pregnant women regarding gestational diabetes mellitus

A small proportion (29.0%) of participants were knowledgeable with a mean score of 7.3 (± 2.1) with a maximum possible score of 12. Most of them (86.16%) did not know of GDM and 83.90% of them were not aware that high blood sugar is a sign and symptom of GDM (Table 2). This was also revealed by one of the pregnant women during the FGD.

“For diabetes disease I heard that one urinates a lot and blood sugars are low.” (FGD-Pregnant Woman 2)

**Table 2:** Frequency distribution of knowledge responses regarding gestational diabetes mellitus among pregnant women (*N* = 354) at Kinango District Hospital, 2019

Variables	Yes		No	
	Number	Proportion (%)	Number	Proportion (%)
*Do you know gestational diabetes mellitus?*	49	(13.84)	305	(86.16)
*Is GDM a condition of high sugar level in blood?*	53	(14.97)	301	(85.03)
*What are the common risk factors of GDM?*				
Older age	17	(4.80)	337	(95.20)
Family history of diabetes mellitus	66	(18.64)	288	(81.36)
Pregnancy	26	(7.34)	328	(92.66)
Lack of exercise	26	(7.34)	328	(92.66)
Poor diet	42	(11.86)	312	(88.14)
*What are the signs and symptoms of GDM?*				
High blood sugar	57	(16.10)	297	(83.90)
Frequent urination	85	(24.01)	269	(75.99)
Excessive thirst	71	(20.06)	283	(79.94)
Excessive hunger	68	(19.21)	286	(80.79)
Feeling of weakness	73	(20.62)	281	(79.38)
Slow healing of cuts and wounds	78	(22.03)	276	(77.97)
Frequent vaginal, bladder and skin infections	24	(6.78)	330	(93.22)
Blurred vision	52	(14.69)	302	(85.31)
*What methods are available for the control and management of GDM?*				
Insulin injection	58	(16.38)	296	(83.62)
Tablets and capsules	102	(28.81)	252	(71.19)
Regular exercise	54	(15.25)	300	(84.75)
Healthy diet practice	60	(16.95)	294	(83.05)
Weight reduction	46	(12.99)	308	(87.01)
Monitoring of baby during ANC visits by HCW	73	(20.62)	281	(79.38)
*What are the complications of GDM?*				
Abortion	42	(11.86)	312	(88.14)
Congenital anomalies (e.g., heart defects)	37	(10.45)	317	(89.55)
Premature birth	65	(18.36)	289	(81.64)
Macrosomia (big baby)	32	(9.04)	322	(90.96)
Pre-eclampsia (hypertension in pregnancy)	75	(21.19)	279	(78.81)
Antepartum hemorrhage (bleeding in pregnancy)	71	(20.06)	283	(79.94)
Difficult labor	69	(19.49)	285	(80.51)
Cesarean section	69	(19.49)	285	(80.51)

#### Factors associated with being knowledgeable about gestational diabetes mellitus among pregnant women

In multivariate logistic analysis secondary education, hearing about DM, family history of DM, and history of previous C/S were significantly associated with GDM knowledge (Table 3).

**Table 3:** Bivariate and multivariate logistic regression analyses indicating associations between various variables and gestational diabetes mellitus knowledge levels among pregnant women (*N* = 354) at Kinango District Hospital, 2019

Maternal variable	COR^1^	95% CI	*P*-value	AOR^2^	95% CI	*P*-value
*Age(categories)*						
< 24 (Ref)						
25–34	1.85	1.13–3.05	0.015			
35–44	0.89	0.39–2.03	0.779			
> 44	1.63	0.14–18.5	0.694			
*Gestational age*						
Less than 24 weeks (Ref)						
24–28 weeks	1.27	0.61–2.64	0.530			
29–42 weeks	0.70	0.33–1.48	0.353			
*Parity*						
Zero (Ref)						
One to two	1.14	0.62–2.12	0.672			
Three to four	0.74	0.37–1.49	0.400			
Five or more	0.70	0.31–1.61	0.403			
Highest level of education*						
Never attended school (Ref)						
Primary	1.44	0.83–2.48	0.190	1.69	0.93–3.04	0.083
Secondary	6.27	2.54–15.49	0.000	5.31	2.04–13.8	0.001
Post-secondary	10.78	3.16–36.72	0.000	5.26	1.43–19.3	0.012
Source of income*						
Housework/informal employment (Ref)						
Business	1.67	0.84–3.33	0.147			
Agriculture	0.69	0.14–3.34	0.650			
Formal employment	6.25	1.87–20.89	0.003			
Ever heard about diabetes mellitus?*						
Yes (Ref)						
No	0.15	0.07–0.30	0.000	0.21	0.10–0.44	0.000
Family history of diabetes mellitus*						
Yes (Ref)						
No	0.23	0.12–0.47	0.000	0.40	0.18–0.87	0.021
History of previous still birth*						
Yes (Ref)						
No	0.22	0.06–0.78	0.018			
*Birth weight of previous child*						
Low birth weight (< 2.5 kg) (Ref)						
Normal birth weight (2.5–4 kg)	0.54	0.19–1.58	0.264			
High birth weight (≥ 4 kg)	0.75	0.05–10.23	0.829			
No previous birth weight	0.79	0.26–2.44	0.683			
History of previous Cesarean section*						
Yes (Ref)						
No	0.14	0.04–0.44	0.001	0.19	0.54–0.65	0.008
*Number of antenatal care visits*						
Zero (Ref)						
One	1.02	0.46–2.25	0.969			
Two	0.76	0.34–1.70	0.497			
Three	0.62	0.30–1.30	0.206			
Greater than or equal to 4	0.56	0.26–1.18	0.128			

*Statistically significant variables

^1^crude odds ratio

^2^adjusted odds ratio

#### Common sources of gestational diabetes mellitus information among pregnant women

One-third of the participants (33.33%) claimed to have heard about GDM from a health facility. This was also revealed by the pregnant women during the FGD. The other common source of information among the few knowledgeable pregnant women was family and neighbors (25.49%), radio (22.88%), television (14.38%) and newspaper (3.92%).

“I heard in the Hospital [Kinango District Hospital] when I came for antenatal […], talks of diabetes disease that affects all people [Diabetes Mellitus] and also in the pregnant […].” (FGD-Pregnant Woman 8).

During the HCW interviews, it was highlighted that pregnant women receive daily health talks on various health topics, including GDM, before an ANC visit.

“Every morning we give health talks before ANC […], we teach topics like Diabetes Mellitus, Gestational Diabetes, Hypertension, Nutrition, Hygiene, they are many.” (KII- Health Care Worker 2).

### Attitudes

Attitude level of pregnant women towards gestational diabetes mellitus at Kinango District Hospital**.**

The portion of participants who had good attitude was 46.98% with a mean score of 20.4 (± 2.2) with a maximum possible score of 25. A greater proportion of the pregnant women (58.19%) agreed on avoiding too much sugar consumption; while 52.26% participants agreed that physical activity can prevent the risk of GDM (Table 4). They were willing to attend ANC for the sake of a healthy pregnancy. But pregnant women interviewed revealed that they were given limited dietary information during ANC health talks.

“We were being told about a healthy diet for the sake of the health of baby during antenatal.” (KII-Pregnant Woman 3)

“The specific food I was told to use for a healthy diet is vegetables, most likely pig weed, beans, fish, eggs, liver, milk, cabbage and potatoes. […]. I am very willing to practice a healthy diet which is important, but it is tough because of one’s earnings.” (KII-Pregnant Woman 1).

**Table 4:** Frequency distribution of attitudes towards gestational diabetes mellitus among pregnant women (*N* = 354) at Kinango District Hospital, 2019

Variable	Response option									
	Strongly disagree		Disagree		Neutral		Agree		Strongly agree	
	*N*	(%)	*N*	(%)	*N*	(%)	*N*	(%)	*N*	(%)
Do you think you should be educated on GDM?	0	(0)	0	(0)	33	(9.32)	153	(43.22)	168	(47.46)
Do you think all pregnant women should be screened for GDM?	0	(0)	0	(0)	31	(8.76)	168	(47.46)	155	(43.79)
Do you think you should follow avoiding consumption of too much sugar?	1	(0.28)	5	(1.41)	47	(13.28)	206	(58.19)	95	(26.84)
Do you think physical activity can prevent the risk of GDM?	1	(0.28)	23	(6.50)	87	(24.58)	185	(52.26)	58	(16.38)
Do you think gestational diabetes mellitus complications on both pregnancy and outcomes may be prevented if blood glucose is well controlled?	1	(0.28)	8	(2.26)	103	(29.10)	196	(55.37)	46	(12.99)

The IDIs among health care workers revealed that they disseminated GDM information from in-service training knowledge, but it was highlighted that there was a need for training on updated GDM guidelines.

“I have never received any in service training on gestational diabetes […], we need seminars and trainings” (KII- Health Care Worker 1)

#### Factors associated with good attitude level towards gestational diabetes mellitus among pregnant women

In multivariate logistic analysis; post-secondary education, hearing about DM, attending at least one ANC visit were significantly associated with good GDM attitude (Table 5).

**Table 5:** Bivariate and multivariate logistic regression analyses indicating associations between various variables and gestational diabetes mellitus attitude levels among pregnant women (*N* = 354) at Kinango District Hospital, 2019

Maternal variable	COR^1^	95% CI	*P*-value	AOR^2^	95% CI	*P*-value
*Age*						
< 24 (Ref)						
25–34	0.99	0.64–1.55	0.979			
35–44	0.91	0.46–1.80	0.782			
> 44						
*Gestational age*						
Less than 24 weeks (Ref)						
24–28 weeks	1.17	0.59–2.31	0.650			
29–42 weeks	0.74	0.38–1.45	0.375			
*Parity*						
Zero (Ref)						
One to two	0.87	0.49–1.54	0.63			
Three to four	0.70	0.37–1.32	0.27			
Five or more	0.64	0.31–1.33	0.23			
Highest level of education*						
Never attended school (Ref)						
Primary	1.39	0.88–2.20	0.162	1.44	0.89–2.32	0.139
Secondary	1.51	0.65–3.53	0.341	1.39	0.57–3.39	0.466
Post-secondary	9.82	2.12–45.4	0.003	8.27	1.73–39.42	0.008
*Source of income*						
Housework/informal employment (Ref)						
Business	1.07	0.55–2.08	0.831			
Agriculture	1.19	0.34–4.19	0.789			
Formal employment	2.67	0.80–8.88	0.108			
Ever heard about diabetes mellitus?*						
Yes (Ref)						
No	0.49	0.31–0.78	0.003	0.48	0.29–0.80	0.004
*Family history of diabetes mellitus*						
Yes (Ref)						
No	0.64	0.32–1.28	0.207			
*History of previous still birth*						
Yes (Ref)						
No	0.49	0.14–0.72	0.267			
*Birth weight of previous child*						
Low birth weight (< 2.5 kg) (Ref)						
Normal birth weight (2.5-4 kg)	3.47	0.96–12.58	0.059			
High birth weight (≥ 4 kg)						
No previous birth weight	4.20	1.10–15.95	0.035			
*History of previous Cesarean section*						
Yes (Ref)						
No	0.58	0.20–1.65	0.304			
Number of antenatal care visits *						
Zero (Ref)						
One	3.19	1.43–7.11	0.005	3.66	1.61–8.30	0.002
Two	1.34	0.60–2.97	0.477	1.61	0.70–3.70	0.258
Three	2.40	1.18–4.88	0.016	3.06	1.44–6.50	0.004
Greater than or equal to 4	1.79	0.87–3.67	0.116	2.01	0.94–4.29	0.071

*Statistically significant variables

^1^crude odds ratio

^2^adjusted odds ratio

### Practices

#### Practice levels of pregnant women towards gestational diabetes mellitus at Kinango District Hospital

Out of 354 participants, 60.17% of them had good practices with a mean score of 13.0 (± 1.9) with a maximum possible score of 19, 70.90% of them claimed to practice 30–60 min of daily physical exercise frequently (Table 6).

**Table 6:** Frequency distribution of practices towards gestational diabetes mellitus among pregnant women (*N* = 354) at Kinango District Hospital, 2019

Variable	Response option									
	Not at all		Less frequent		Not sure		Frequent		Very frequent	
	*N*	%	*N*	%	*N*	%	*N*	%	*N*	%
Do you consume sugary and fatty foods?	106	(29.94)	167	(47.18)	21	(5.93)	50	(14.12)	10	(2.82)
Do you do 30–60 min of daily physical activity? E.g., walking, house chores	0	(0)	3	(0.85)	6	(1.69)	251	(70.90)	94	(26.55)
Do you check your sugar levels during antenatal clinic?	108	(30.51)	59	(16.67)	153	(43.22)	26	(7.34)	8	(2.26)

The KIIs among pregnant women revealed that they had limited information regarding the specific exercises suitable for pregnancy.

“I was told to walk at times, […]and to fetch water, wash clothes and utensils.” (KII-Pregnant woman 4)

The HCW KII revealed the type of practice that the health professionals conveyed to the pregnant women.

“Normally the physical exercise we encourage is only walking and some light household duties.” (KII-Health Care Worker 3)

#### Factors associated with good practice levels towards gestational diabetes mellitus among pregnant women at Kinango District Hospital

In multivariate logistic analysis, gestational age of 24–28 weeks, hearing about DM, birth weight of previous child (≥ 4 kg) and one ANC visit were significantly associated with good GDM practices (Table 7).

**Table 7:** Bivariate and multivariate logistic regression analyses indicating associations between various variables and GDM practice levels among pregnant women (*N* = 354) at Kinango District Hospital, 2019

Maternal variable	COR^1^	95% CI	*P*-value	AOR^2^	95% CI	*P*-value
*Age*						
< 24 (Ref)						
25–34	1.06	0.67–1.68	0.791			
35–44	0.79	0.40–1.58	0.512			
> 44	0.33	0.03–3.70	0.367			
Gestational age*						
Less than 24 weeks (Ref)						
24–28 weeks	1.85	0.89–3.82	0.098	1.80	0.77–4.20	0.173
29–42 weeks	0.51	0.25–1.01	0.053	0.65	0.25–1.67	0.372
*Parity*						
Zero (Ref)						
One to two	0.53	0.29–0.98	0.042			
Three to four	0.66	0.34–1.27	0.214			
Five or more	0.74	0.34–1.58	0.434			
*Highest level of education*						
Never attended school (Ref)						
Primary	1.05	0.66–1.67	0.836			
Secondary	1.10	0.46–2.61	0.836			
Post-secondary	1.03	0.34–3.07	0.961			
*Source of income*						
Housework/informal employment (Ref)						
Business	0.86	0.44–1.68	0.656			
Agriculture	0.63	0.18–2.24	0.480			
Formal employment	0.74	0.24–2.26	0.598			
Ever heard about diabetes mellitus?*						
Yes (Ref)						
No	0.26	0.17–0.42	0.000	0.28	0.17–0.47	0.000
Family history of diabetes mellitus*						
Yes (Ref)						
No	0.45	0.21–0.99	0.046			
*History of previous still birth*						
Yes (Ref)						
No	0.33	0.07–1.53	0.156			
Birth weight of previous child*						
Low birth weight(< 2.5 kg) (Ref)						
Normal birth weight (2.5-4 kg)	1.48	0.52–4.19	0.466	0.99	0.32–3.06	0.986
High birth weight (≥ 4 kg)	2.29	0.17–30.96	0.534	2.10	0.15–29.7	0.583
No previous birth weight	3.22	1.05–9.92	0.042	2.26	0.68–7.51	0.183
*History of previous Cesarean section*						
Yes (Ref)						
No	0.54	0.17–1.72	0.294			
Number of antenatal care visits*						
Zero (Ref)						
One	4.15	1.72–10.03	0.002	6.05	2.31–15.8	0.000
Two	2	0.91–4.39	0.084	3.65	1.50–8.90	0.004
Three	1.44	0.73–2.86	0.293	3.75	1.61–8.71	0.002
Greater than or equal to 4	0.78	0.39–1.55	0.470	2.24	0.90–5.59	0.084

*Statistically significant variables; ^1^crude odds ratio; ^2^adjusted odds ratio

## Discussion

Our study revealed that, of the 354 pregnant women, 71% of them were not knowledgeable, 53% of them had relatively poor attitude, and 60% had good practices regarding GDM.

### Knowledge

Evidently, knowledge regarding GDM was found to be low in this study; only 29.10% pregnant women reported being knowledgeable. The knowledge scores in our study were lower than those found in the rural area of Southeastern Ethiopia (52.5%) [31]. This may be explained by comparing the sources of information between our study and the Ethiopia study. Among knowledgeable pregnant women, their common GDM information source was reported to be from the health facility (33.33%), followed by the family and neighbors (25.49%); while the radio (22.88%), television (14.38%) and newspaper (3.92%) accounted for the least sources of GDM information. The latter was also highlighted in the pregnant women’s FGD as the pregnant women pointed out that they were given health talks during ANC visits that included both DM and GDM. Further evidence from the HCW interviews revealed that daily health talks were conducted on many health topics such as DM, GDM, hypertension, and hygiene. In contrast to our study, the Ethiopia study found a greater knowledge score and reported media as the major source of information [31]. Healthcare workers may need to come up with innovative complementary means of spreading GDM information, e.g., via media. Astonishingly, it was of great concern to find out from our study that most pregnant women (83.90%) were unaware of the fact that high blood sugar is a sign and symptom of GDM (Table 2), considering that pregnant women had heard of DM during ANC visits. A study conducted in the four regions of Kenya reported similar results [7]. The latter study attributed the low level of knowledge, regarding signs and symptoms, to the limited availability of comprehensive health promotion programs for most non-communicable diseases. This may also be a possible explanation for the low level of knowledge specifically regarding GDM signs and symptoms in our study too. Hence, there is an urgent need for healthcare workers to scale up on GDM awareness programs for pregnant women, through the creation of culturally specific means of dissemination of GDM information such as the use of visual aid leaflets, banners, and posters; as well as conducting plays during ANC and areas of mass gatherings like the markets and churches [7, 21].

### Attitudes

Only 46.98% of participants in this study had a good attitude toward GDM; attendance of at least one ANC visit was associated with a fourfold increase in good attitude as compared to those that had zero ANC visits in our study (Table 5). The attendance of at least one ANC visit was 85.88%, which was slightly below the expected level among the majority of pregnant women, who were expected to have attended at least one ANC visit based on their gestational age (87.86%). Specifically, in this context, women with a gestational age greater than 24 weeks (87.86%) were required to attend at least one ANC visit (Table 1). Hence, our results were incongruent with the required number of ANC visits as per gestational age, highlighted in both the World Health Organization (WHO) 2002 Focused ANC Model; as well as the recent WHO 2016 ANC Model [32, 33]. This reduced number of ANC visits as per requirement for gestational age needs to be addressed as more pregnant women need to be encouraged to attend ANC to obtain necessary information for a healthy pregnancy that may influence their attitude and will have an impact on their practices. The pregnant women KIIs pointed out the importance of ANC visits as they were advised on a healthy diet, walking, and continued house chores. Thus, it is commendable that pregnant women were given some information regarding lifestyle modification during ANC. In addition, the pregnant women also pointed out that they acquired knowledge about DM from the daily health talks during ANC at Kinango District Hospital, thus this may have influenced their GDM practices. A recent study by Mukona et al. [34], reported on the importance of ANC attendance with emphasis on the initial visit as this may allow for GDM education and possible early interventions to prevent GDM complications [34]. This was also supported by other studies that highlighted the importance of ANC attendance in the reduction of maternal–child health complications [35, 36]. Basically, during ANC, healthcare workers disseminate information regarding DM, GDM, and self-care practices, e.g., healthy diet and exercise that enhance health awareness and interest among individuals thereby influencing their practice levels [17]. Thus, the Kenyan Ministry of Health may also consider using the media via already existing health programs as a means of encouraging earlier and complete ANC attendance to improve health awareness that may affect GDM prevention practices among pregnant women.

### Practices

Unexpectedly, our results pointed out that 60.17% pregnant women had good GDM practices despite having poor knowledge regarding GDM. This was a similar finding of a study conducted across four regions in Kenya by Kiberenge et al. [7] that revealed good practices among individuals with poor knowledge in Coastal Kenya. The authors attributed the latter results to indigenous knowledge [7]. Senanayake, described indigenous knowledge as a unique knowledge confined to a particular culture or society passed on from generation to generation [37]. As such indigenous knowledge may have influenced the good practices of individuals both in our study and that of Kiberenge et al. [7], despite studies having revealed poor knowledge. We believe that there should be more studies investigating indigenous knowledge regarding GDM. Because indigenous knowledge is often rooted in a deep understanding of the environment and the interconnections between different aspects of life, it can provide valuable insights and approaches to encourage changes in practices and should be further investigated. Additionally, it has the potential to make promotional healthcare programs more accessible and relevant to communities. The KIIs among pregnant women confirmed that they were educated about some form of physical exercise and diet during ANC. However, our study disclosed that pregnant women were not given precise information on specific food portions and specific exercises during pregnancy. This gap in specific lifestyle modification information needs to be addressed. Thus, there is a need for the healthcare workers at Kinango District Hospital to educate pregnant women on the use of locally available and affordable produce for a healthy diet during ANC. Healthcare workers also need to educate pregnant women on specific pregnancy exercises that are feasible and affordable for pregnant women to carry out in rural areas. The latter action is vital for a healthy pregnancy; and most importantly, the International Federation of Gynecology and Obstetrics (FIGO) has emphasized that lifestyle modification is key for the prevention and control of GDM in pregnancy [38]. On the other hand, it was revealed in the KIIs with healthcare workers, that there has never been any in-service training for GDM specifically targeting midwives and senior registered nursing officers who attend to pregnant women in ANC. However, it was evident that healthcare workers gave daily health talks based on their pre-service training knowledge. This highlighted the urgent need to plan for HCW training by the district management team, especially for those that mostly attend to pregnant women. The need for GDM training was also expressed in studies conducted in the UK [39, 40]. Considering that pregnant women receive most of their information from healthcare workers, there is a need for healthcare workers to obtain updated information on specific diets and exercises to convey comprehensive lifestyle modification information to pregnant women during ANC. Thus, it is crucial for healthcare workers to provide better lifestyle advice to pregnant women during ANC, such as precise physical activity and diet during pregnancy to improve self-care practices. All in all, lifestyle modification will help in the prevention of GDM and, in the long run, the prevention of non-communicable diseases such as diabetes mellitus.

### Strengths and limitations

The strength of this study lies in its focus on an area with limited existing literature. To the best of our knowledge, the level of KAPs regarding GDM among pregnant women at Kinango District Hospital has not been previously studied. Additionally, no studies have investigated the factors associated with GDM KAP among pregnant women at Kinango District Hospital. The results of our study may stimulate further research and contribute to the evidence base, thereby assisting policymakers in evaluating the effectiveness of current GDM policies in Kenya.

A major limitation of our study was that we were unable to collect blood glucose samples due to ethical clearance constraints. Thus, we were unable to determine the prevalence of GDM at Kinango District Hospital. Furthermore, this study was conducted at one health facility in Kinango as such the results obtained may not be representative of all the pregnant women in Kinango. Moreover, the convenient sampling may have exposed our study to selection bias.

### Recommendations

Due to limitations, there is a need for a follow-up study in Kinango targeting the collection of blood glucose to determine the prevalence of GDM. Furthermore, a community-based random recruitment strategy for a follow-up study, to improve the generalizability of results, may be considered. In addition, in-service training on up-to-date guidelines, for existing healthcare workers who see pregnant women during ANC is highly recommended. Nonetheless, the relevant authorities may consider integrating specific GDM prevention activities into already existing health promotion activities targeting pregnant women in Kinango.

## Conclusion

Generally, it is apparent that despite the low level of knowledge and relatively good attitude regarding GDM, pregnant women at Kinango District Hospital had good GDM practices. This may be attributed to the daily health talks that are conducted by healthcare workers at the facility during ANC, as well as indigenous knowledge among pregnant women. Hence, GDM information spread may be enhanced by being incorporated into locally available health promotion programs, to improve the level of GDM knowledge, attitude, and practices among pregnant women. This will contribute towards the prevention of GDM and ultimately the prevention of non-communicable diseases such as diabetes mellitus.

## Data Availability

The datasets during and/or analyzed during the current study are available from the corresponding author on reasonable request.
